# Inclisiran—Safety and Effectiveness of Small Interfering RNA in Inhibition of PCSK-9

**DOI:** 10.3390/pharmaceutics15020323

**Published:** 2023-01-18

**Authors:** Łukasz Wołowiec, Joanna Osiak, Anna Wołowiec, Aleksandra Wijata, Elżbieta Grześk, Mariusz Kozakiewicz, Joanna Banach, Alicja Nowaczyk, Jacek Nowaczyk, Grzegorz Grześk

**Affiliations:** 1 Department of Cardiology and Clinical Pharmacology, Faculty of Health Sciences, Collegium Medicum in Bydgoszcz, Nicolaus Copernicus University, 87-100 Toruń, Poland; 2 Department of Geriatrics, Division of Biochemistry and Biogerontology, Collegium Medicum in Bydgoszcz, Nicolaus Copernicus University, 87-100 Toruń, Poland; 3 Department of Pediatrics, Hematology and Oncology, Collegium Medicum in Bydgoszcz, Nicolaus Copernicus University, 87-100 Toruń, Poland; 4 Department of Organic Chemistry, Faculty of Pharmacy, Collegium Medicum in Bydgoszcz, Nicolaus Copernicus University, 87-100 Toruń, Poland; 5 Department of Physical Chemistry and Physicochemistry of Polymers, Faculty of Chemistry, Nicolaus Copernicus University, 7 Gagarina St., 87-100 Toruń, Poland

**Keywords:** inclisiran, PCSK9, siRNA, dyslipidemia, atherosclerosis

## Abstract

Dyslipidemia is listed among important cardiovascular disease risk factors. Treating lipid disorders is difficult, and achieving desirable levels of LDL-cholesterol (LDL-C) is essential in both the secondary and primary prevention of cardiovascular disease. For many years, statins became the basis of lipid-lowering therapy. Nevertheless, these drugs are often insufficient due to their side effects and restrictive criteria for achieving the recommended LDL-C values. Even the addition of other drugs, i.e., ezetimibe, does not help one achieve the target LDL-C. The discovery of proprotein convertase subtilisin/kexin type 9 (PCSK9) discovery has triggered intensive research on a new class of protein-based drugs. The protein PCSK9 is located mainly in hepatocytes and is involved in the metabolism of LDL-C. In the beginning, antibodies against the PCSK9 protein, such as evolocumab, were invented. The next step was inclisiran. Inclisiran is a small interfering RNA (siRNA) that inhibits the expression of PCSK9 by binding specifically to the mRNA precursor of PCSK9 protein and causing its degradation. It has been noticed in recent years that siRNA is a powerful tool for biomedical research and drug discovery. The purpose of this work is to summarize the molecular mechanisms, pharmacokinetics, pharmacodynamics of inclisiran and to review the latest research.

## 1. Introduction

Dyslipidemia, especially elevated levels of LDL cholesterol (LDL-C), is mentioned as a substantial risk factor for cardiovascular disease. A high concentration of LDL-C is regarded as a great risk of atherosclerosis, including coronary artery disease. The main causal and modifiable atherosclerotic cardiovascular disease (ASCVD) risk factors are blood apolipoprotein-B-containing lipoproteins (especially LDL-C), high blood pressure, cigarette smoking, and diabetes mellitus (DM). Modern cardiovascular pharmacotherapy, especially for diseases of atherosclerotic etiology, is extremely multidirectional [1,2]. Among them, the most important is the intervention at the level of the vascular endothelium [3,4], modifying the release and production of mediators and, secondarily, the functions of the receptor systems of signal conducting systems [3,5]. Another direction of influence is the influence on the function of blood platelets responsible for the progression of the disease and its exacerbations [6,7,8], as well as the growth of atherosclerotic plaques [9,10]. Cardiovascular diseases (CVD) are responsible for over 4 million deaths annually in Europe [11]. The number of people diagnosed with primary hypercholesterolemia and mixed dyslipidemia has doubled in recent years [12]. Statins have invariably been the first-line drugs in the treatment of lipid disorders for many years. Besides, ezetimibe (ATC code: C10AX09 [13]) is also widely used. Despite intensive lipid-lowering therapy with a high-dose statin and ezetimibe, many patients still do not achieve the recommended LDL-C levels, often due to side effects [14]. The new target is the PCSK9 (proprotein convertase subtilisin/kexin 9) protein [15]. PCSK9 is a protein located mainly in hepatocytes and plays a role in the metabolism of LDL-C. The discovery of the PCSK9 protein has led to the creation of a new group of drugs—PCSK9 inhibitors, which include two monoclonal antibodies—evolocumab (ATC code: C10AX13 [16]) and alirocumab (ATC code: C10AX14 [17]). The combination of a PCSK9 inhibitor, high-intensity statin treatment, and ezetimibe reduces LDL-C by approximately 85% [18]. An even newer alternative approach to PCSK9 relies on RNA interference. Inclisiran (ATC code: C10AX16 [19]), a low molecular weight compound, interferes with RNA (siRNA), inhibiting PCSK9 expression. The mechanism of this process involves specifically binding to the mRNA precursor of PCSK9 protein and its further degradation [20]. Unlike monoclonal antibodies, which only lower the extracellular levels of PCSK9, inclisiran lowers the intra- and extracellular levels of PCSK9 [21]. It has been shown to decrease LDL-C levels up to approximately 50% depending on the dose [22]. So far, it has been noticed that inhibitors of PCSK9 do not increase the risk of diabetes or muscle pain and myopathy—as is the case with statin therapy [23]. An additional advantage is the method of administering the drug—at intervals of several months, and the administration of the drug under the supervision of medical personnel increases the effectiveness and allows one to objectively assess whether the patient is taking the drug. Recently, we can observe the results of many studies on new and promising drugs used in cardiology, for instance, levosimendan (ATC code: C01CX08 [24]) in heart failure or drugs acting on the PCSK9 protein used in dyslipidemia [25]. The main objective of this work is to discuss molecular mechanisms, pharmacokinetics, and pharmacodynamics and to review the latest research on inclisiran.

## 2. Dyslipidemia

Cardiovascular disease is the leading cause of death among adults. People with lipid disorders have approximately double the risk of developing CVD compared to people who have normal total cholesterol levels [26]. Familial hypercholesterolemia (FH) is associated with an even greater risk of developing CVD at a younger age; early diagnosis and the implementation of treatment reduce the number of CVD-related events and premature death [27]. Modifiable risk factors for hyperlipidemia include physical inactivity, a diet rich in saturated and trans fats, obesity, and smoking. Secondary causes include diseases such as high blood pressure, bile duct obstruction, type 2 diabetes, chronic kidney disease, and hypothyroidism [26]. The American College of Cardiology/American Heart Association’s (ACC/AHA) ASCVD risk calculators may be useful in evaluating a patient’s individual ASCVD risk. However, these calculators have some limitations in that they can be applied to a specific group of white and African-American men and women who are between the ages of 40 and 79. Therefore, these calculators will not be suitable for other age groups or other races [28]. Applying appropriate pharmacotherapy for dyslipidemia, encouraging public education, increasing the availability of lipid tests, spreading greater awareness of dyslipidemia complications as well as continuously updating and developing dyslipidemia guidelines are key issues that help improve the treatment of dyslipidemia [29,30].

Currently, the therapeutic goal in lipid-lowering therapy is to achieve the target LDL-C. Based on the 2019 ESC guidelines, low-risk patients may consider LDL-C < 3.0 mmol/L (<116 mg/dL) as a target. For moderate-risk patients, LDL-C and LDL-C < 2.6 mmol/L (<100 mg/dL) should be considered. For primary prevention in very high-risk patients, a reduction of ≥50% from baseline in LDL-C and LDL-C level of <1.4 mmol/L (<55 mg/L) is recommended as the target. For secondary prevention in very high-risk patients, a reduction of ≥50% from baseline in LDL-C and an LDL-C level of <1.4 mmol/L (<55 mg/dL) is recommended as the target. In patients with ASCVD who experience a second vascular event (not necessarily of the same type as the first) within 2 years despite treatment with a statin at the maximum tolerated dose, an LDL-C level <1.0 mmol/L (<40 mg/dL) should be considered [31].

It has been shown that statins, despite their proven effectiveness in reducing LDL cholesterol, show considerable variability in response to these drugs. Moreover, some studies show that half of the people starting statin therapy discontinue it within a year, and in patients with persistently high LDL cholesterol and high cardiovascular risk, the rate of CVD events remains high, necessitating the modification of therapy [32,33]. PCSK9 inhibitors are an additional way to treat lipid disorders associated with elevated LDL cholesterol. By binding to the PCSK9 protein, they inhibit the degradation of the LDL receptor. Consequently, a greater number of LDL receptors contribute to the reduction of LDL-C plasma concentration [34,35].

Inclisiran is a small interfering RNA (siRNA) used in LDL-lowering therapy. Thanks to the possibility of using a highly specific endogenous mechanism to regulate gene expression, inclisiran therapy has become a promising therapeutical option in the treatment of dyslipidemia [36,37].

## 3. PCSK9

PCSK9 (initially named neural apoptosis-regulated convertase-1) is a key protein regulating the level of circulating low-density lipoprotein cholesterol (LDL-C) [38]. The work published in 2003, which gave rise to research on the PCSK9 protein, was a discovery made in French families—in people without mutations in the LDLR or apolipoprotein B gene but with very high levels of LDL-C [39]. This protein is most highly expressed in hepatocytes. In addition to hepatocytes, the expression of PCSK9 was noted in the cells of the small intestine, and its much smaller amounts were also noted in the thymus, lungs, kidneys, and spleen [40].

LDL-C is cleared from the circulation by the LDL receptor (LDLR). The pro-protein convertase subtilisin/kexin 9 (PCSK9) enhances the degradation of LDLRs in endosomes/lysosomes, resulting in an increase in circulating LDL-C [41]. Indeed, PCSK9 is a serine protease belonging to the proprotein convertase family, and essential for the metabolism of LDL particles by inhibiting LDLR recirculation to the cell surface with the consequent upregulation of LDLR-dependent LDL-C levels [42]. Some studies also suggest a role for PCSK9 in increasing tumor metastasis [43,44]. As it turns out, the role of PCSK9 goes beyond the regulation of circulating lipid levels, and its inhibition may have positive pleiotropic effects in patients at increased cardiovascular disease risk [26]. One study also showed that PCSK9 levels are associated with the progression of atherosclerosis, as reflected by the total area of atherosclerotic plaques, regardless of plasma LDL-C concentration [27].

## 4. Anti-PCSK9 Antibodies

Research on the PCSK9 protein contributed to the development of antibodies against the PCSK9 protein—evolocumab, alirocumab, and bococizumab. Unlike evolocumab and alirocumab, which are monoclonal antibodies, bococizumab is a humanized antibody (containing a few percent of mouse proteins). It was evaluated in the SPIRE-1 and SPIRE-2 studies; it caused a noticeable decrease in the hypolipemic effect after several months related to the formation of antibodies against the drug molecule, but a frequent occurrence of allergic reactions at the injection site was also observed, and for this reason, the study was suspended [45]. Currently, the FDA and EMA have approved evolocumab and alirocumab. They are antibodies against PCSK9 and drugs with a low potential for side effects. The main adverse events were injection site pain, back pain, nasopharyngitis, headache, upper respiratory tract infection, and flu-like symptoms (7.5%) [23]. PCSK9 inhibitors do not have an adverse effect on glucose metabolism and the increase in the number of new cases of diabetes. This has been demonstrated in the FOURIER (evolocumab), OSLER-1 (evolocumab), and ODDYSEY LONG (alirocumab) studies, among others [46,47,48,49]. The blockade of PCSK9 by evolocumab significantly reduced cardiovascular risk in diabetic and non-diabetic patients. One of the major limitations of statin therapy is muscle pain and the risk of myopathy. The DESCARTES study (Durable Effect of PCSK9 Antibody Compared with Placebo Study) showed that side effects (an increase in creatine kinase above normal level and muscle pain) were observed at the same level in both groups (placebo vs. evolocumab) [50]. It can also be concluded that previous studies have not shown that therapy with a PCSK9 inhibitor has a harmful effect on cognitive functions [51,52]. Can we also expect such a range of safety in the case of a drug with the siRNA mechanism—inclisiran?

## 5. Small Interfering RNA—Short History and Mechanism

Eukaryotic mRNAs are molecules that live longer than those found in bacteria (for mammals this time is several hours). Due to the differences in half-life, scientists wondered what the processes responsible for mRNA degradation were and how they were controlled. In 1998, Fire et al. discovered the mechanism of RNA interference, which revolutionized the understanding of gene regulation. They established that double-stranded RNA molecules are the silencing effectors in Caenorhabditis elegans [53]. In their experiment, they used single-stranded RNA (ssRNA) or double-stranded RNA (dsRNA) to disrupt muscle function. The results they obtained indicate that the occurrence of an effective knockdown led to phenotypic muscle twitching of Caenorhabditis elegans. Moreover, the only way to increase the efficiency of ssRNA was to simultaneously inject ssRNA with the antisense strand, which suggests that hybridization of ssRNA to dsRNA is a precondition for gene silencing [54].

The target RNA silencing molecule must be double-stranded, which excludes cellular mRNAs but includes viral genomes, many of which are double-stranded RNAs in their native state, or double-stranded RNAs are intermediate in their replication. Double-stranded RNA is recognized by binding proteins, which create a binding site for a ribonuclease called Dicer that cuts the molecule into short interfering RNA (siRNA) 21–28 nucleotides long [55]. Thanks to this, the viral genome is inactivated. However, a question arises: what happens if the viral genes have already been transcribed? It might seem that silencing RNA will not protect the cell from damage. Therefore, the breakthrough discovery was the demonstration of the existence of a second step in the interference process directed at viral mRNA. The siRNA molecules produced by cleavage are target sites for the formation of the RISC complex (RNA-induced silencing complex) [56]. They are separated into single strands; one strand is degraded, and the other strand of each siRNA base pairs with viral mRNAs present in the cell. RISC complex contains an RNA binding protein of the Argonaut family and a cutting and therefore silencing mRNA nuclease [57,58].

Since research on C. elegans, it has been shown that RNA interference occurs in all eukaryotes with single exceptions, for example, in S. cerevisiae. The interference process itself has been associated with phenomena including RNA degradation. For example, the movement of some types of mobile elements is mediated by double-stranded RNA molecules. This is one way for eukaryotic organisms to prevent the mass multiplication of transposons in their genomes.

**Figure 1:**
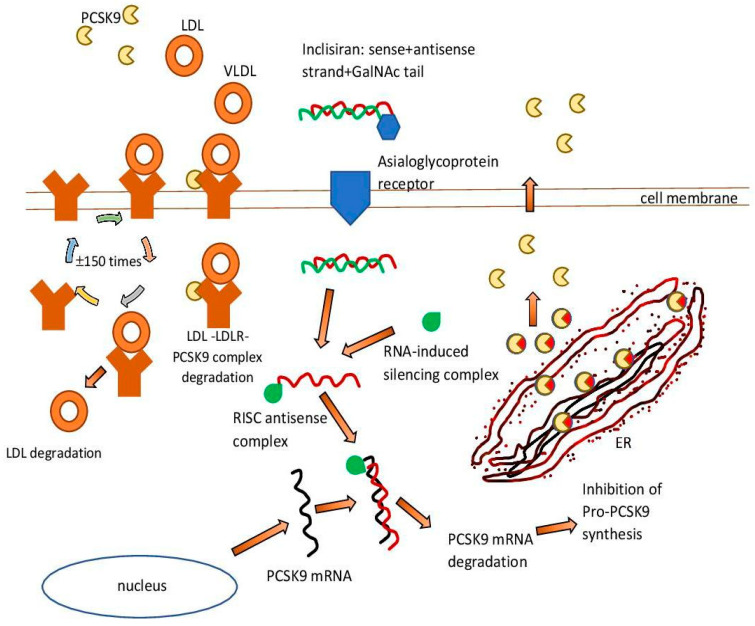
Inclisiran—mechanism of action. ER—endoplasmic reticulum. GAlNAc—N-acetylgalactosamine. LDL—low-density lipoprotein. LDLR—low-density lipoprotein receptor. PCSK9—proprotein convertase subtilisin/kexin 9. RISC—RNA-induced silencing complex. VLDL—very low-density lipoprotein.

RNAi therapies represent a huge opportunity in the treatment of many diseases. siRNA has the ability to target and silence virtually any gene of interest. Consequently, siRNA can be a powerful tool for biomedical research and drug discovery [59]. Therefore, during the development of low molecular weight inhibitors targeting protein-resistant oncogenes, examples such as RAS and MYC posed a serious challenge [60,61]. siRNA-based therapy can turn the tide of many debilitating diseases, as siRNAs are easily engineered, and strong siRNA identification can be done in weeks compared to years for drug development [62]. Another advantage is that strong siRNA sequences are usually active at extremely low (picomolar) concentrations, and they can be designed for any gene of interest with the appropriate tools [62,63] (Figure 1).

An example of such a therapeutic intervention is the drug inclisiran [64]. It is a double-stranded cholesterol-lowering siRNA, linked on the coding strand to the triantenary N acetylgalactosamine (N-acetylgalactosamine, GalNAc). The aim of this is to facilitate its uptake by hepatocytes. The RNA interference mechanism in hepatocytes is used by inclisiran to direct catalytic mRNA breakdown to PCSK9. This action intensifies the expression of LDL-C receptors and their recycling on the surface of hepatocytes and consequently enhances LDL-C uptake and reduces circulating LDL-C levels [65].

## 6. siRNA Drugs

Research conducted for nearly 20 years on siRNA finally contributed to the approval by the FDA in November 2018 of the first drug of the siRNA group—patisiran (ATC code: N07XX12 [19]). It was a groundbreaking event that opened the door for new drugs to be introduced to the market. Patisiran is a drug used in the polyneuropathy of hereditary TTR-dependent amyloidosis (hATTR) [66,67]. In a 12-month analysis of the ongoing study-OLE study, conducted in 211 patients, patisiran appeared to maintain efficacy with an acceptable safety profile in patients with hereditary TTR-dependent amyloidosis with polyneuropathy [68]. In one of the studies, it was additionally shown that patisiran can stop or reverse the progression of cardiac symptoms of hATTR amyloidosis [69]. The most common treatment-related adverse event was mild or moderate infusion-related reactions [68].

Another drug was givosiran (ATC code: A16AX16 [19]). Givosiran is a drug approved for the treatment of patients with acute porphyria (AHP) [70]. Among patients with acute intermittent porphyria, those who received givosiran had a significantly lower rate of porphyria attacks and better scores on many other symptoms of the disease than those who received a placebo [71,72]. Adverse effects on the liver (increased levels of aminotransferases) and kidneys (changes in creatinine levels) have been recorded as side effects, among others [71].

Lumasiran (ATC code: A16AX18 [73]) was the third consecutive small interfering ribonucleic acid approved by the EMA and FDA. It is indicated for the treatment of a rare genetic disease—primary hyperoxaluria type 1. The effectiveness of this drug has been confirmed in the ILLUMINATE-A, ILLUMINATE-B, and ILLUMINATE-C studies [74,75,76]. Next was inclisiran, a drug registered in the treatment of ASCVD (atherosclerotic cardiovascular disease) and HeFH (heterozygous familial hypercholesterolemia)—this drug will be discussed in more detail later in the article. The last drug approved this year by the EMA and FDA was vutrisiran—a drug used in hereditary transthyretin amyloidosis (ATTRv) with polyneuropathy. In studies, vutrisiran significantly improved many disease-relevant outcomes compared to placebo, with an acceptable safety profile [77]. Research is underway to introduce further siRNA drugs—nedosiran, fitusiran, tivanisiran, and olpasiran [78,79,80,81].

The table below shows the drugs from the siRNA group that have approved so far by the EMA/FDA (Table 1).

## 7. Pharmacokinetic and Pharmacodynamic Properties

The systemic exposure to inclisiran increased approximately dose-proportionally over the range of 24 mg to 756 mg after a single subcutaneous administration. At a dose of 284 mg, plasma concentrations reached maximum concentrations approximately 4 h post-dose, with a mean Cmax of 509 ng/mL. In vitro, at appropriate clinical plasma concentrations inclisiran is 87% protein bounded. After the subcutaneous administration of a single 284-mg dose, the apparent volume of distribution was ≈500 L [82].

Based on preclinical data, inclisiran has been shown to have high uptake and selectivity for hepatocytes [83]. Inclisiran is mainly metabolized by non-specific nucleases to inactive shorter nucleotides [64]. The drug is almost completely cleared from the circulation within 24 h after subcutaneous injection [84]. The terminal elimination half-life of inclisiran is approximately 9 h and accumulation does not occur with repeated dosing. It is estimated that sixteen percent of inclisiran is cleared by the kidneys [82]. Regardless of renal impairment, inclisiran has a short plasma half-life (5–10 h) [85].

The effects on PCSK9 and LDL cholesterol levels were sustained for at least 180 days after initiation of treatment, with little variability over a period of 6 months after receiving the first dose [86]. Doses of 300 mg or more (in single or multiple doses) significantly lowered PCSK9 and LDL-C levels for at least 6 months. There was also a reduction in PCSK9 to 83.8% and LDL cholesterol to 59.7% at a dose of 300 mg [86]. The subcutaneous administration of inclisiran provided tissue-specific delivery and efficacy, leading to the potent and dose-dependent inhibition of PCSK9 gene expression. The study shows that 1 mg/kg was an approximate effective dose, causing 50% inhibition. For comparison, 6 mg/kg was an approximate effective dose, causing 80% inhibition of PCSK9, and then, the maximum inhibitions of PCSK9 and LDL-C were 85% and 68% [87]. Moreover, the study showed that the inclisiran-treated patients had lower non-HDL cholesterol, lipoprotein(a), and apolipoprotein B levels. They also had higher levels of HDL cholesterol [88].

In patients with moderate hepatic impairment, the pharmacokinetic exposure of inclisiran was up to two-fold higher compared to patients with normal hepatic function, while the pharmacodynamic effect was relatively unchanged. Studies have shown that inclisiran is generally safe and well tolerated in patients with mild or moderate hepatic impairment, without the need for dose adjustment. The pharmacodynamic effects and safety profile of inclisiran were similar in subjects with normal and impaired renal function. There is no need to adjust the inclisiran dose in these patients [89].

Therefore, inclisiran can probably be used safely even in patients with advanced kidney disease (CrCl level, 15–29 mL/min). However, it should be noted that people with acute kidney disease, those who had undergone kidney transplantation, and those requiring hemodialysis were excluded from the study—further studies are certainly needed in these groups of patients [90].

## 8. Safety and Side-Effect Profile

All adverse events were mild to moderate in severity and, importantly, did not cause discontinuation of the study in any of the participants. The most commonly reported adverse events are cough, musculoskeletal pain, headache, back pain, diarrhea, and nasopharyngitis [91,92]. One study participant taking a statin showed an asymptomatic increase in GGTP and ALT, with no increase in bilirubin—the increase in enzymes subsided after stopping the statins. In addition, a few study participants developed a delayed, mild, self-limited injection site rash as well as mild, reversible discoloration from the injection site rash. No changes in the corrected QT interval (QTc) were observed [86]. Based on contemporary scientific reports, one can conclude that inclisiran is a well-tolerated LDL-C-decreasing agent. However, as with any new substance, the potential off-target effects and the long-term safety of the drug should be closely monitored. A study that assesses long-term tolerance to inclisiran administration is the ORION-3 study (extension study of the phase 2 ORION-1 trial). The long-awaited results were published a few days ago in Lancet. The above-mentioned study outcomes showed that twice-yearly inclisiran provided sustained reductions in LDL-C and PCSK9 levels and was well tolerated for 4 years [93]. Patients receiving inclisiran in ORION-1 received inclisiran during ORION-3 as well, whereas patients receiving placebo in ORION-1 received evolocumab for up to 1 year and then transitioned to inclisiran for the remainder of the study. The treatment-emergent adverse events, which were possibly related to the study’s medication, occurred in 79 (28%) of 284 patients (in the inclisiran-only arm)—the most common were injection site reaction (16 patients [5.6%]), injection site erythema and injection site pain (12 patients [4.2%]). A hepatic enzyme increase occurred in 4 patients (1,4%), as well as muscle spasms. Treatment-emergent serious adverse events, possibly related to the study drug (as reported by the investigator), occurred in 4 patients and included sinus tachycardia, acute cholecystitis (in a patient known with gallstones), hepatic fibrosis (in a patient with fatty liver disease) and hepatic enzyme increased (in a patient with chronic hepatitis C and high alcohol intake) [93]. In the ORION-3 study, each reported side effect was summarized in detail in the supplementary material to Lancet’s article. Another study examining the efficacy, safety, and tolerability of long-term dosing of inclisiran is the ORION-8 study. The study will include more than 3000 participants previously involved in the ORION-3, ORION9, ORION 10, and ORION-11 studies. The estimated data collection completion time is the end of 2023 [94]. As rightly noted, inclisiran could be a very interesting option in pregnancy, as it can be used just before and immediately after pregnancy for about 9 months between injections [95]. However, as a precaution—due to a lack of adequate research, it is recommended to avoid using inclisiran during pregnancy.

## 9. Clinical Trials

The effectiveness and safety of inclisiran are evaluated by the ORION program, which consists of worldwide studies in a specific population, including high-risk atherosclerotic cardiovascular disease (ASCVD) and diagnosed ASCVD or familial hypercholesterolemia (FH) [96]. Two randomized, single-blind, placebo-controlled studies of phase 1 in healthy adult volunteers showed mean long-term dose-related reductions in circulating LDL-C and PCSK9. The safety and tolerability profile of inclisiran was similar to the placebo [97]. The first double-blind, placebo-controlled, multicenter phase 2 study was the ORION-1 study. In 501 patients who had known ASCVD or were at a high risk of ASCVD, inclisiran was administered in multiple increasing doses. The most beneficial effect in reducing LDL-C and PCSK9 levels were achieved with the two-dose inclisiran (300 mg) regimen, with a reduction of 52.6% and 69.1% after 180 days. Serious adverse events occurred in 11% of patients taking inclisiran and 8% of patients taking a placebo. During the observation of the study participants up to one year after the first dose of inclisiran, a sustained reduction of LDL-C within the first year was demonstrated [98]. The effectiveness and safety of inclisiran were assessed depending on the occurrence of diabetes—inclisiran was associated with marked declines in LDL-C in the groups of patients without and with diabetes [88].

The evaluation of the effectiveness of inclisiran is based on 3 pivotal clinical trials: ORION-9, ORION-10, and ORION-11, which are multicenter, double-blind, randomized, placebo-controlled phase 3 trials. The results of the studies show that the administration of inclisiran in a two-dose schedule compared to the placebo leads to a sustained and marked reduction of LDL-C in patients with elevated LDL-C [99]. The ORION-9 study included patients with genetic or clinical features of HeFH and LDL-C ≥ 100 mg/dL [100], while ORION-10 and ORION-11 included patients with ASCVD and LDL-C ≥ 70 mg/dL. The ORION-11 study additionally included patients with an ASCVD risk equivalent, including diabetes mellitus type 2 (DM2), HeFH, or a 10-year risk of a cardiovascular event ≥ 20%, assessed according to the Framingham Risk Score for CVD or equivalent [101]. In the ORION-9 study, 300 mg of inclisiran or placebo was administered subcutaneously on the first day, third month, ninth month, and fifteenth month. In the 17-month study, a 39.7% decrease in LDL-C was achieved in the inclisiran group, while an increase in LDL-C by 8.2% was observed in the placebo group. A significant reduction in LDL-C was noted in all FH genotypes, with a similar frequency of serious adverse events in both groups [99]. The ORION-10 and ORION-11 studies included 1561 and 1617 patients, respectively, who were administered subcutaneous inclisiran at a dose of 300 mg or placebo on day 1, month 3, and then every 6 months until the end of the observation period. After 510 days of observation, inclisiran lowered LDL-C by 52.3% in the ORION-10 study and by 49.9% in the ORION-11 study. In both trials, the incidence of serious adverse events was similar [101]. This year’s published results of the ORION-3 study showed that inclisiran, when administered in addition to the maximum tolerated standard lipid-lowering therapy, is effective and safe and causes a remarkable reduction in LDL-C levels in patients from South Africa with high cardiovascular risk [102].

ORION-4 is a key study in the entire ORION program and enrolled approximately 15,000 patients with pre-existing ASCVD and assessed the effect of inclisiran on major adverse cardiac events (MACE). The subjects were randomized into the inclisiran group or placebo group. This ORION study included patients > 50 years of age and ≥1 of the following criteria: prior ischemic stroke, peripheral arterial disease, or myocardial infarction. ORION-4 is a study aimed at assessing the 5-year risk of MACE in study participants. The study is scheduled to be completed in 2026 [103].

VICTORION-2P is an ongoing study to evaluate the benefits of inclisiran in major adverse cardiovascular (MACE) events in participants with known cardiovascular disease (CVD). It is estimated that the first outcomes of the VICTORION-2P study will be published in 2027 [104] (Table 2).

## 10. Familial Hypercholesterolemia (FH)

Familial hypercholesterolemia (FH) is a monogenic, dominantly inherited dyslipidemia that contributes to premature CVD due to lifelong elevated plasma LDL-C levels. The diagnosis of FH is based in most cases on the clinical picture. Dutch Lipid Clinic Network criteria or, for example, WHO criteria are often used [105,106]. Moreover, in the diagnostic process, genetic testing is used. There are homozygous and heterozygous forms. In the homozygous form, the course is rapid—patients can experience symptoms of ischemic heart disease in childhood or young adulthood.

In 2021, the FDA approved evinacumab as an add-on treatment for patients 12 years of age and older with homozygous familial hypercholesterolemia (HoFH), a genetic condition that causes very high cholesterol. It is a fully humanized anti-ANGPTL3 antibody. The ELIPSE-HoFH study evaluated the therapeutic efficacy of evonacumab in 65 patients with HoFH. The mean baseline plasma LDL-C level, despite intensive lipid-lowering treatment, was 255 mg/dL. After 24 weeks, a significant reduction in plasma levels of LDL cholesterol (as much as 47%), and moreover—total cholesterol, triglycerides, non-HDL, apoB, apoC-III, and Lp(a) was demonstrated [107].

However, in patients with the heterozygous variant of HeFH disease, the course is less dynamic, but despite this, the disease still significantly increases the cardiovascular risk. The main study focusing on HeFH patients is the above-mentioned ORION-9 study. A significant reduction in LDL-C was noted in all FH genotypes, with similar rates of serious adverse events in both groups (inclisiran vs. placebo). Inclisiran is approved for the treatment of HeFH [100].

The ORION-5 trial is a phase 3, multicenter, double-blind, placebo-controlled trial that will evaluate the safety, tolerability, and efficacy of inclisiran in individuals with homozygous familial hypercholesterolemia (HoFH) [108].

## 11. Olpasiran

Recent studies and guidelines draw attention to lipoprotein (a) (Lp (a)) and its impact on the risk of ASCVD [105,109,110]. According to the 2019 guidelines, Lp(a) measurement should be considered in every adult at least once in a lifetime to identify individuals with congenital very high Lp(a) levels >180 mg/dL (>430 nmol/L) who are at a lifetime risk for ASCVD may be comparable to the risk associated with HeFH (recommendation class IIa) [1].

To date, no drugs targeting Lp(a) directly are available for clinical use, but olpasiran is during clinical trials. Olpasiran is a synthetic, double-stranded, N-acetylgalactosamine-conjugated siRNA designed to directly inhibit the translation of LPA messenger RNA in hepatocytes and potently reduce plasma Lp(a) concentration. In the phase 1 study, the primary endpoints were safety and tolerability, with the secondary endpoints being changes in Lp(a) concentrations and olpasiran pharmacokinetic parameters. Participants tolerated single doses of olpasiran well and experienced Lp(a) reductions of 71–97% with effects lasting for several months after the administration of a dose of 9 mg or more [111].

In comparison, a single administration of monoclonal antibodies against PCSK9 (evolocumab) resulted in a 14% decrease in Lp(a) [112]. In a phase 2 study conducted on 281 patients, it was shown that olpasiran therapy significantly reduced the concentration of lipoprotein(a) in patients with diagnosed atherosclerotic cardiovascular disease [113]. Phase 3 trials are planned, with the primary objective of comparing the effect of olpasiran treatment with a placebo on the risk of death from ischemic heart disease, myocardial infarction, or urgent coronary revascularization in participants with atherosclerotic cardiovascular disease (ASCVD) and elevated Lp(a); however, the recruitment of study participants has not yet started, and the estimated date of completion of the study is December 2026 (ClinicalTrials.gov Identifier: NCT05581303) [114].

## 12. Pediatric Population

The number of clinical trials conducted in the pediatric population is limited. This is the main reason why so many therapeutic options commonly used in adults are only off-label options in the group of children and adolescents. The treatment of lipid disorders is no exception. For many years, the therapeutic procedure was very conservative. It was limited to dietary treatment and physical activity therapy. Currently, the use of statins and ezetimibe is not in doubt in children with homozygous and heterozygous hypercholesterolemia.

Currently, the therapy with 3-hydroxy-3-methyl-glutaryl-coenzyme A reductase inhibitors (statins) is a cornerstone of the therapy. This kind of pharmacology leads to an increase in LDL receptors’ density and a decrease in secondary LDL concentration [115]. For children at the age 8 years and older rosuvastatin and pravastatin have been approved, whereas atorvastatin, lovastatin, simvastatin, and fluvastatin (ATC code: C10AA04 [116]) have been approved for use at 10 years of age [117,118]. Pharmacotherapy with statins should take into account not only the recommended age of the child in relation to clinical trials but also the expected reduction in LDL cholesterol. The next step is to extend the therapy with ezetimibe [118].

Extremely interesting is the option of treatment with PCSK9 inhibitors. The HAUSER-RCT study showed that 24 weeks of PCSK9 inhibitor evolocumab in pediatric patients with heterozygous familial hypercholesterolemia was safe and improved lipid parameters compared to placebo. Prolonged observation HAUSER-OLE was an 80-week, single-arm, open-label extension of HAUSER-RCT and a randomized controlled trial that was conducted at 46 centers in 23 countries. Pediatric patients aged 10–17 years with heterozygous familial hypercholesterolemia who completed 24 weeks of monthly treatment with subcutaneously administered placebo or 420 mg evolocumab in HAUSER-RCT with no serious treatment-emergent adverse events were eligible to enroll in HAUSER-OLE. All patients received open-label subcutaneous evolocumab 420 mg monthly with background statins with or without ezetimibe for 80 additional weeks. The study confirmed that evolocumab was safe, well tolerated, and led to sustained reductions in LDL cholesterol in pediatric patients with heterozygous familial hypercholesterolemia [119,120,121]. Results of trials suggest that for pediatric patients with heterozygous familial hypercholesterolemia, additional lipid-lowering treatments are needed [122].

In that condition, the last therapeutic option with inclisiran usage was interesting. The objective of studies ORION-13 and ORION-16 is to investigate the efficacy, safety, and tolerability of inclisiran in adolescents diagnosed with homozygous familial hypercholesterolemia (ORION-13) or heterozygous familial hypercholesterolemia (ORION-16). The studies are two-part (1-year double-blind inclisiran vs. placebo/1 year open-label inclisiran) multicenter trials including adolescents aged 12 to 18 years diagnosed with familial hypercholesterolemia with an additional small number of children diagnosed with homozygous familial hypercholesterolemia. Looking at the pharmacokinetics of this therapeutic option, it can be concluded that after confirming the safety and effectiveness of inclisiran, we will obtain a great therapeutic tool also for use in children [123,124].

## 13. Summary

Treatment of lipid disorders is difficult and achieving desirable LDL-C levels is essential in both secondary and primary prevention of cardiovascular disease. Dyslipidemia is one of the established risk factors for cardiovascular disease. Frequently, LDL-C targets cannot be achieved with statins and ezetimibe, among other things, due to the side effects of statins. Therefore, it seems that therapy with PCSK9 inhibitors is an inevitable step in achieving the target LDL-C values. Inclisiran is a new promising drug that inhibits the action of mRNA transduction of the PCSK9 gene. The effectiveness of inclisiran and its safety profile have been proven in published ORION studies. The unique dosing regimen (starting dose, next dose after three doses, then every 6 months thereafter) and its long-lasting effect help to overcome the challenges of patient non-compliance. Moreover, the studies conducted so far show a favorable adverse event profile of inclisiran. The results of ongoing trials are expected to shed more light on the impact of improving treatment outcomes in ASCVD patients and reducing cardiovascular events. In addition to inclisiran, another lipid-lowering drug from the siRNA group is under clinical trials—olpasiran. It is likely that even more drugs from this group will be developed in the coming years. It seems that lipid-lowering therapy will take on a completely new dimension.

## Figures and Tables

**Table 1:** siRNA drugs approved by EMA/FDA.

Drug Name	EMA	FDA	Indications
patisiran	August 2018	August 2018	hATTR amyloidosis in patients with stage 1 or stage 2 polyneuropathy
givosiran	March 2020	November 2019	AHP
lumasiran	November 2020	November 2020	PH 1
inclisiran	December 2020	December 2021	ASCVD, HeFH
vutrisiran	July 2022	June 2022	hATTR amyloidosis in patients with stage 1 or stage 2 polyneuropathy

EMA—European Medicines Agency, Food and Drug Administration, hATTR—hereditary transthyretin amyloidosis, AHP—acute hepatic porphyria, PH 1—primary hyperoxaluria type 1, ASCVD—atherosclerotic cardiovascular disease, HeFH—heterozygous familial hypercholesterolemia.

**Table 2:** Key clinical trials of inclisiran.

	Participants	Design	Outcome
Completed trials			
ORION-1 phase 2	501 participants (253 between 18 and 65 years, 248 ≥ 65 years); ASCVD or ASCVD risk equivalents and elevated LDL-C	Multicenter, double-blind, placebo-controlled study. One dose (200, 300, or 500 mg on day 1) or 2 doses (100, 200, or 300 mg on days 1 and 90) of inclisiran sodium or placebo.	Mean percentage change from baseline in LDL-C on Day 180: 38.4% with single dose of inclisiran; 52.6% with two-dose starting regimen of inclisiran
ORION-3 phase 2	382 participants; ASCVD or ASCVD risk equivalents and elevated LDL-C who have completed ORION-1 study	4-year open-label extension study of the placebo-controlled, phase 2 ORION-1 trial. Patients receiving inclisiran in ORION-1 received inclisiran sodium, whereas patients receiving placebo in ORION-1 received evolocumab for up to 1 year and then transitioned to inclisiran for the remainder of the study.	Twice-yearly inclisiran provided sustained reductions in LDL-C and PCSK9 concentrations and was well tolerated over 4 years.
ORION-10 phase 3	1561 participants (630 between 18 and 65 years, 931 ≥ 65 years); ASCVD and elevated LDL-C	Double-blind, randomized, placebo-controlled study. Patients received either inclisiran (284 mg) or placebo, administered subcutaneous on day 1, day 90, and every 6 months thereafter over a period of 540 days.	At day 510, inclisiran reduced LDL-C levels by 52.3% with corresponding time-adjusted reductions of 53.8%. Adverse events and serious adverse events were similar in the two groups.
ORION-11 phase 3	1617 participants (733 between 18 and 65 years, 884 ≥ 65 years); ASCVD or ASCVD risk equivalents and elevated LDL-C;	Double-blind, randomized, placebo-controlled study. Patients received either inclisiran (284 mg)) or placebo subcutaneous on day 1, day 90, and every 6 months thereafter over a period of 540 days.	At day 510, inclisiran reduced LDL-C levels by 49.9% with corresponding time-adjusted reductions of 49.2%. Adverse events and serious adverse events were similar in the two groups.
Ongoing trials			
ORION-4 phase 3	15,000 participants; pre-existing ASCVD	Patients will receive inclisiran 300 mg or placebo on day 1, after 3 months and then every 6 months.	Estimated primary completion date: July 2026. Estimated study completion date: December 2049. The purpose of this study is to find out if inclisiran safely lower MACE in people with pre-existing ASCVD.
ORION-8 phase 3	3275 participants (actual enrollment)	Inclisiran sodium 300 milligrams will be administered as a single subcutaneous injection od Day 1, 90, then every 180 days to Day 990.	Estimated primary completion date: 31 December 2023. The purpose of this extension study is to evaluate the efficacy, safety and tolerability of long-term dosing of inclisiran.
VICTORION-2P	15,000 participants (estimated enrollment)	Patients will receive inclisiran 300 mg or placebo.	Estimated primary completion date: 13 October 2027 Estimated Study Completion Date 13 October 2027

ASCVD—Atherosclerotic Cardiovascular Disease, CVD—cardiovascular disease, HeFH—heterozygotus familial hypercholesterolemia, LDL-C—LDL-cholesterol, MACE—major adverse cardiovascular events.

## Data Availability

Not applicable.
